# Deep Inspiration Breath Hold to Reduce Cardiovascular Disease Risk for Breast Radiotherapy: Challenges and Opportunities

**DOI:** 10.1016/j.jaccao.2024.08.009

**Published:** 2024-11-19

**Authors:** Shanshan Gao, Jian Zhang, Beina Hui, Weibin Hu, Yongkai Lu

**Affiliations:** a The First Affiliated Hospital of Xi'an Jiaotong University, Xi’an, Shaanxi, China

The recent study by Busschaert et al^1^ on the cost-effectiveness of deep inspiration breath hold (DIBH) for left-sided breast radiotherapy offers significant insights into its cardioprotective benefits. The investigators convincingly highlight the ability of DIBH to reduce the mean heart dose and the subsequent risk of cardiovascular disease in breast cancer patients.^1^ However, the study raises several points that warrant further discussion.

Firstly, although DIBH is cost-effective within the Belgian health care system, its broader applicability needs consideration. The incremental cost-effectiveness ratio of €14,023 per quality-adjusted life-year fits within the willingness-to-pay threshold in Belgium but may not apply in lower-income countries. In these settings, the additional costs of DIBH might be prohibitive, given resource constraints and the need to prioritize interventions with immediate benefits. Future studies should assess the cost-effectiveness of DIBH in diverse economic contexts to ensure global relevance.^2^

Secondly, the operational challenges of DIBH are significant. The study noted a 12.48% reduction in maximum throughput, potentially leading to longer waiting times and delayed treatment initiation. This issue is particularly concerning in high-volume centers. The impact of these delays on patient outcomes, especially cancer control, needs further exploration. Strategies such as workflow optimization or incorporating artificial intelligence to streamline DIBH could mitigate these challenges and should be investigated.^3^

Finally, although the reduction in mean heart dose with DIBH is notable, the study does not fully consider the variability of an individual patient anatomy and its effect on the benefits of DIBH. In clinical practice, refining patient selection criteria for DIBH could ensure that those most likely to benefit are prioritized.^4^ Integrating personalized dosimetric planning could enhance the efficacy of DIBH, making it more targeted and effective.^5^

In conclusion, Busschaert et al^1^ have contributed valuable data to the field of radiotherapy, highlighting the potential of DIBH to improve long-term cardiovascular outcomes in breast cancer patients. However, the challenges associated with its implementation, particularly in diverse economic and clinical settings, must be addressed to fully realize its benefits. Further research should aim to refine patient selection, optimize workflow, and evaluate the global cost-effectiveness of DIBH.

## References

[1] Busschaert S.-L., Kimpe E., Gevaert T., De Ridder M., Putman K. (2024). Deep inspiration breath hold in left-sided breast radiotherapy. JACC CardioOncol.

[2] Darby S.C., Ewertz M., McGale P. (2013). Risk of ischemic heart disease in women after radiotherapy for breast cancer. N Engl J Med.

[3] Keel G., Savage C., Rafiq M., Mazzocato P. (2017). Time-driven activity-based costing in health care: a systematic review of the literature. Health Policy.

[4] Macrie B.D., Donnelly E.D., Hayes J.P. (2015). A cost-effective technique for cardiac sparing with deep inspiration-breath hold (DIBH). Physica Medica.

[5] Koene R.J., Prizment A.E., Blaes A., Konety S.H. (2016). Shared risk factors in cardiovascular disease and cancer. Circulation.

